# Neural substrates of sexual arousal in heterosexual males: event-related fMRI investigation

**DOI:** 10.1186/s40101-016-0089-3

**Published:** 2016-03-08

**Authors:** Ji-Woo Seok, Jin-Hun Sohn, Chaejoon Cheong

**Affiliations:** 1 Department of Psychology, Brain Research Institute, Chungnam National University, Koong-dong 220, Yusung-gu, Daejeon, Korea; 2 Bioimaging Research Team, Division of Bioconvergence Analysis, Korea Basic Science Institute, eongudanji-ro 162, Ochang, Cheongju, Korea

**Keywords:** Sexual arousal, Hemodynamic response, Event-related paradigm, Globus pallidus, Putamen

## Abstract

**Background:**

Sexual behavior is an important role for the survival of species. The advancement of brain imaging methods has enabled the understanding of the brain mechanism related to sexual arousal. The previous studies on the brain mechanism related to sexual arousal have mostly conducted on block design paradigm.

**Methods:**

Despite its requirement for stricter experimental control, the event-related paradigm is known to be more efficient in detecting instant emotional and cognitive responses. The paradigm also enables the observation of hemodynamic responses through time. Therefore, this study used the event-related fMRI to examine the brain activation in various areas associated with sexual arousal as well as changes in hemodynamic responses with time.

**Results:**

Strong activations were observed in the various areas associated with sexual arousal comprised of various factors: (1) activation areas related to cognitive factors: the occipital lobe and parietal lobe; (2) activation areas related to emotional factors: the thalamus and amygdala; (3) activation areas related to motivational factors: the anterior cingulate gyrus, orbitofrontal cortex, and insula; and (4) activation areas related to physiological factors: the precentral gyrus, putamen, and globus pallidus. We also identified the activation of the putamen and globus pallidus that were not well observed in previous block design studies. In the result of the hemodynamic response, the neural activity in those areas showed more transient aspects of the hemodynamic responses relative to the neural activity of other areas.

**Conclusions:**

These results suggested that the event-related paradigm is better at detecting the neural activity of the brain regions, which tend to appear suddenly, but disappear soon.

## Background

Sexual arousal is a multi-dimensional experience including cognitive, emotional, physical, and behavioral aspects. That is, sexual arousal is the physical characteristics or behavioral reactions which appear as the result of an interaction and competition between positive psychological responses (e.g., sexual attraction or hedonia by charms) and negative responses (e.g., anxiety, sense of guilt, shyness) after the perception of a given object [1–3]. Previous studies on sexual arousal have mainly focused on sexual response, including reaction mechanisms of neural vascular, autonomic nervous, and hormonal systems [4–6]. With the recent development of brain imaging technology that makes possible the examination of brain function, including the cognitive and emotional processes in a noninvasive way, this has helped us to understand the neural mechanisms of cognition and emotion with respect to sexual arousal.

Recently, a lot of research has been conducted on the neural mechanism of sexual stimuli visually presented using fMRI. Previous studies have shown that sexually arousing stimuli are linked to diverse neural networks such as the parietal lobe, temporo-occipital lobe, frontal lobe, cerebellum, insula, anterior cingulate gyrus, amygdala, and striatum related to emotional, cognitive, motivational, and physiological components [7–13]. However, as mentioned above, most of the previous studies on the brain mechanism for sexual arousal have adopted the block design. Research based on the event-related design has been performed relatively little.

The block design is used to identify the brain areas which are linked to a certain stimulus. The block design presents an experimental condition and baseline condition in turn for dozens of seconds. By presenting the experimental condition (e.g., task condition) and baseline condition alternatively and repeatedly, it is supposed to detect relevant brain area to task condition. The relevant brain area can be found by measuring the blood oxygen level-dependent (BOLD) signals which increase when the stimulation condition is presented but disappear when the baseline condition is shown. The block design is known to be better at detecting brain areas related to a certain stimulus compared to the event-related design [14, 15]. It is also easier to design the stimulus presentation paradigm and to compare the differences between each stimulus when the block design is applied. Further, artifacts or noises can be eliminated more easily through the visual analysis of time series data [14, 15]. However, the block design has some weak points. For instance, since the stimuli of similar conditions are presented for dozens of seconds, the problem of habituation may appear. Also, as the time for showing a stimulus gets longer, the BOLD signals decrease [16].

The event-related design for fMRI is developed based on event-related potential studies, that is the functional brain responses occurring during events (i.e., presented stimulus or behavioral trials) [17]. When the event-related design is applied to experiments, more complex design and analysis are required [18]. For example, the number of trials should be considered to compensate for the weak signal intensity and the random jittering of the trials also should be applied to ensure that the activation signals did not overlap. This information should also be reflected in the analysis, which requires a more complicated process [19]. The event-related design is a relatively recent development compared to the block design. In this method, the BOLD signal that is accompanying each event, i.e., the presentation of the stimulus, can be observed by aligning and averaging the imaging data obtained before and after the presentation time of the stimulus in terms of time. This approach has the advantage of being able to detect a temporal change in the hemodynamic response unlike block design method. In recent years, the detection power increases in the event-related design as in the block design does via the development of imaging techniques with high resolution (i.e., high signal-to-noise ratio (SNR)); studies using event-related design methods have been growing [17]. It requires the strict experimental control of stimulus presentation time, the interstimulus interval, and the presentation order of stimulus. Also, since this information should be reflected in the analysis, complex analysis is required in the method [18]. As the stimulus presentation is randomized in the event-related design, confounds caused by stimulus order predictability can be reduced. In the event-related design, the occurrence of unconsecutive stimuli cannot be predictable and stimulus presentation length is also shorter than that in the block design [18]. Therefore, transient brain response such as emotional and physiological processing can be measured better in event-related design compared to block design [18]. A few studies using this event-related design on sexual arousal have been conducted [13, 20]. However, these studies investigated temporal changes of brain signals associated with sexual arousal by measuring either only the partial regions and not the whole brain or the examination of the whole brain without observing the hemodynamic response. Therefore, the study is to examine the brain areas and hemodynamic response related to sexual arousal by applying event-related fMRI. We hypothesized that the study can detect activations in the brain areas that were not well detected due to rapid processing of the stimulus and habituation in the prior studies using the event-related fMRI research on sexual arousal. In addition, it is expected to observe the hemodynamic response in the regions by increasing the ISI time.

## Methods

### Subjects

In total, 17 adult males ranging in age from 22 to 29 years participated in the experiment. The participants were all normally sexually functioning, right-handed heterosexuals. Those who are sexually perverted or challenged were excluded. The participants agreed to participate in the experiment of this study after being informed about the content of the experiment.

### Procedure and experimental paradigm

A group of 130 healthy male college students participated in a pre-test to select sexual stimuli for the fMRI study. A total of 237 photos were selected from the International Affective Picture System (IAPS) [21] and Internet searches and were presented to the participants. Images from the Internet searches consisted of photos of porn and naked women. We asked the participants to respond to the question “Did you have the feeling of sexual arousal?” by choosing “yes” or “no” to each stimulus. They were then required to rate the intensity of the sexual arousal on a five-point Likert scale ranging from 1 (least intense) to 5 (most intense). Validity was defined as the percentage of the participants who experienced sexual arousal for each stimulus, and effectiveness was defined as the intensity of the sexual arousal that the participant experienced for each stimulus. As a result of the pre-test, 20 photos (6 IAPS pictures and 14 from the Internet searches) were selected as the sexual stimuli that had 80 % or higher validity and 4 points or higher effectiveness. Twenty sexual stimuli were selected. In addition, 20 photos that did not induce any sexual arousal were chosen as the nonsexual stimuli. The nonsexual stimuli displayed scenes similar in nature to highly arousing images of water sport activity, celebrating a winning victory, and skiing. The stimuli selected from the IAPS and the Internet searches were matched with the sexual stimuli for their level of pleasantness. Mean scores of pleasantness and arousal to sexual stimuli were 5.23 (standard deviation (SD) = 0.36) and 5.17 (SD = 0.31), respectively. Twenty nonsexual stimuli matched with the sexual stimuli for their level of pleasantness (*M* = 5.10, SD = 0.31) and arousal 4.96 (SD = 0.38) were selected. The mean scores of pleasantness and arousal between sexual stimuli and nonsexual stimuli were not significantly different (*t* = −1.18, *p* > 0.05; *t* = −1.99, *p* > 0.05).

In the fMRI experimental paradigm, a brief instruction about the experiment was given for 6 s at the beginning, followed by the presentation of sexual stimulus or nonsexual stimulus, randomly selected, for 5 s each. Each inter-stimulus interval was presented for 7–13 s (average 10 s) to observe the hemodynamic response. The total experiment time was 8 min and 48 s. To prevent the loss of concentration due to the long interval between stimulus presentations, the participants were asked to press the response key whenever a green screen was displayed during the interval (the green screen was randomly displayed 12 times). After completing the fMRI experiment, the participants were required to respond to the following three questions in the psychological assessment. Firstly, they were asked to respond “yes” or “no” regarding whether they felt sexual arousal with each stimulus. Secondly, they were required to rate how intense the sexual arousal was on a Likert scale ranging from 1 (least intense) to 5 (most intense). The participants then were required to report any other emotions that they experienced besides sexual arousal during their exposure to each stimulus.

### Imaging acquisition

A 3.0T Philips MR Scanner was used to acquire images, and the single-shot EPI fMRI scan method was applied to obtain BOLD images. In total, 35 slides were consecutively collected using the image parameter of TR = 2000, TE = 28 ms, a slice thickness of 5 mm with no gap, a 64 × 64 matrix, a FOV 24 × 24 cm, a flip angle = 80°, and an in-plane resolution of 3.75 mm. For the anatomical image of T1 weighted, the FLASH sequence was used.

### Statistical analyses

For the psychological data analysis, a two-tailed paired *t* test was performed using SPSS 22 to compare the frequency (i.e., the number stimuli that evoked sexual arousal out of 20 pictures in each condition; represented as the percentage) and intensity of sexual arousal (i.e., the average level of subjective sexual arousal in each condition) between the sexual and neutral conditions. The fMRI data were analyzed using the Statistical Parametric mapping, version 8 (SPM 8, Wellcome Department of Imaging Neuroscience, London, UK). In the preprocessing stage, the time difference between slice images produced during the acquisition of fMRI images was corrected. Also, to remove the artifact caused by the movement during the experiment, the head movements of the participants were adjusted using 3-D rigid body registration with 6 degrees of freedom (*x*, *y*, *z*, roll, pitch, and yaw). Then, the coregistration and spatial normalization of each participant were done. In order to adjust the fMRI images of each participant to the Montreal Neurological Institute (MNI) coordinate system, the average image of a participant’s fMRI images was matched to the anatomical image of that participant for their coregistration. The adjusted structural image was matched to the MNI coordinate system. Using the normalization parameter produced in the process, the fMRI image was matched to the MNI coordinate system. Lastly, the smoothing of data was performed using a Gaussian kernel, which is the 8 mm in full width at half maximum. The same process was performed for each participant.

After the preprocessing to identify the brain areas activated by sexual arousal, a design matrix consisting of two conditions (i.e., sexual stimulus and nonsexual condition) was produced for each participant. In order to reduce artifacts and noises, diverse regression variables were added during the creation of the design matrix. Specifically, the correction of signal changes due to head movements, i.e., the degree of translation and rotation observed in the process of the correction of the head movements, was included as a regression variable. For the adjustment of low signal recorded in the late phase of the experiment due to habituation, the time modulation option implemented in SPM 8 was used as a regression variable. Then, the contrast was made and compared between the sexual stimuli and nonsexual stimuli to identify the brain areas activated by the sexual arousal of each participant. For the group analysis, one-sample *t* test and paired *t* test were performed. One-sample *t* test was conducted to identify the brain activation during each condition (i.e., sexual arousal condition and nonsexual condition). Consequently, the paired *t* test was performed to examine the brain activation difference between the two conditions, i.e., the brain activation in the sexual arousal condition compared to nonsexual condition. The MNI coordinates of the voxel activated at the *p* value of 0.05 (corrected, false discovery rate (FDR)), which is a significant level in the fMRI study, were applied to find the significant activation in the study. Then, the MNI coordinates were converted into Talairach coordinates to identify the anatomical labeling of the brain activation. Also, to identify the hemodynamic responses of the activated areas, the percent signal change of the regions of interest (ROIs) was extracted in each subject using MarsBaR (http://www.sourceforge.net/projects/marsbar). The coordinates used in the ROI analysis were obtained from the result of one-sample *t* test and were defined as the centering spheres on the peak voxel with a radius of 5 mm.

## Results

### Results of psychological response

The analysis of the psychological responses showed that frequencies of sexual arousal in each condition were 74 ± 7.79 % (the mean ± SD; represented as a percentage) in an experimental condition and 0 ± 0 % in baseline condition. The intensities based on a five-point scale were 2.86 points ± 0.40 (the mean ± SD) in an experimental condition and 0 ± 0 in baseline condition. A paired *t* test indicated that there were differences in the frequency and intensity of sexual arousal between sexual stimuli and nonsexual stimuli (frequency *t* (16) = 29.01, *p* < 0.001; intensity *t* (16) = 39.43, *p* < 0.001).

### Results of brain imaging for group analysis

#### Brain activation responses during nonsexual stimulus presentation

The brain areas activated by the nonsexual stimuli included the cuneus, medial occipital gyrus, lingual gyrus, the fusiform gyrus, hipocampal gyrus (BA 27), posterior cingulate gyrus, and cerebellum (corrected FDR, *p* < 0.05) (Table 1).

#### Brain activation responses during sexual stimulus presentation

When sexual stimuli were presented, the occipital gyrus, the precentral gyrus, the anterior cingulate gyrus (BA 24), hipocampal gyrus (BA 27), thalamus, putamen, claustrum, and cerebellum were activated (corrected FDR, *p* < 0.05) (Table 2).

#### Difference in activation areas between sexual stimuli and nonsexual stimuli

**Fig. 1:**
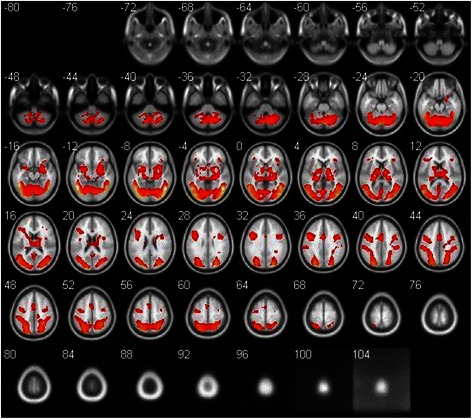
Regions demonstrating significant differences between the sexual stimuli and nonsexual stimuli conditions. Areas are significant at *p* < 0.05, FDR corrected at the cluster level for multiple comparisons across the whole brain

**Fig. 2:**
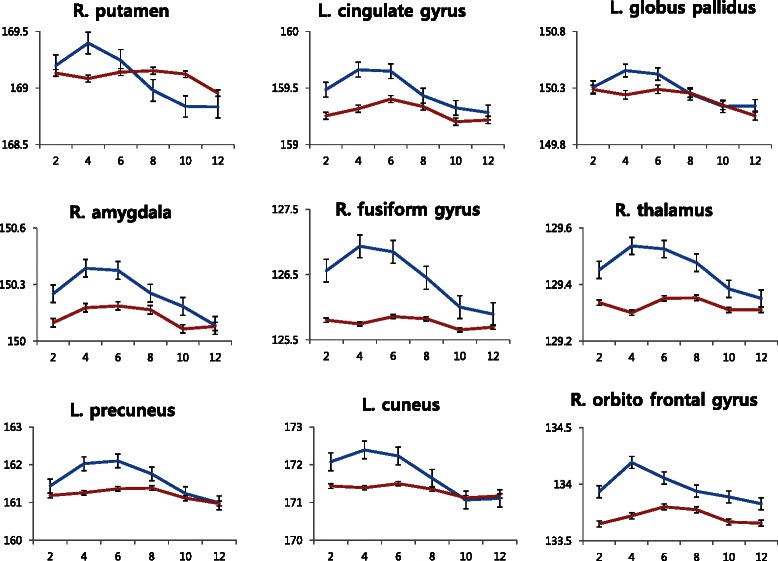
Hemodynamic responses between the sexual stimuli and nonsexual stimuli conditions in the regions of interest. The fitted data are shown as changes in blood oxygen level-dependent signal over time. Values are the mean ± SEM. The *y-axis* and *x-axis* are expressed in arbitrary units and seconds. *R* right, *L* left, *blue*, the response of sexual condition, *red* the response of nonsexual condition

Activation was observed in the bilateral medial occipital gyrus (BA 18, 19), fusiform gyrus (BA 37), precuneus (BA 19), inferior parietal cortex (BA 40), the orbital frontal cortex (BA 47), thalamus, insula, globus pallidus, putamen, and amygdala in response to sexual stimuli compared with nonsexual stimuli (corrected FDR, *p* < 0.05), as shown in the Table 3 and Fig. 1. There was no area which showed greater activity during nonsexual stimuli than sexual stimuli. Figure 2 shows the hemodynamic responses for each condition in the selected ROIs based on the result of the one-sample *t* test. As for the pattern of hemodynamic responses, a smooth curved shape was observed for the canoes, precuneus, fusiform gyrus, thalamus, amygdala, orbital frontal cortex, and anterior cingulate gyrus. In the case of the globus pallidus and putamen, a sharp curve was shown.

## Conclusions

This study identified the brain activation associated with sexual arousal caused by visual stimuli and observed the hemodynamic responses of those areas with time using event-related fMRI. The study results show that the brain areas related to sexual arousal include the orbito frontal cortex, cuneus, precuneus, fusiform gyrus, anterior cingulate gyrus, amygdala, globus pallidus, putamen, and thalamus. The changes in the hemodynamic responses of these areas are shown in Fig. 2.

According to previous studies, the sexual arousal caused by visual stimuli is the result of interactive working on diverse neural mechanisms. This includes the emotional, cognitive, motivational, and physiological mechanisms involved in the processes from the recognition of a stimulus to the response by the somatic nervous system [22, 23]. That is, the cognitive mechanism determines whether a visual stimulus is a sexual stimulus or not, and if recognized as a sexual stimulus, it evaluates its sexual valence. The emotional mechanism is related to the feeling of pleasure due to a sexual stimulus (how much a stimulus is sexually pleasing), while the motivational mechanism is known to be associated with the determination on whether to express sexual arousal as a behavioral response. The physiological mechanism is known to be involved in the physiological responses (responses of the autonomic nervous system and endocrine system) associated with sexual arousal.

The relationship between each mechanism and the brain areas activated by sexual arousal as identified in this study can be explained as follows. The bilateral medial occipital gyri and fusiform gyrus are thought to be linked to the recognition and determination of a stimulus as a sexual stimulus. Based on previous studies that were conducted to identify the brain areas related to the emotions caused by visual stimuli, the occipital gyri and fusiform gyrus were activated more when emotional visual stimuli were presented than when nonsexual stimuli were shown due to increased attention [24–26]. The observed result is also in line with the previous studies investigating sexual arousal induced by visual stimuli, and they reported that the activation of the fusiform gyrus is linked to the function of recognizing a presented stimulus as a sexual stimulus [3, 27].

The activation of the precuneus and the inferior parietal cortex located in the parietal lobe is also thought to be related to the cognitive mechanism. This observation is supported by previous studies of sexual arousal induced by tactile stimuli [28, 29]. According to these studies, the penis enlargement and the intensity of perceived sexual arousal had a positive relationship with the activated size of the parietal lobe, and the activated area of the parietal lobe was the secondary sensation area (BA 40). They reported that the activation of the parietal lobe is likely to be associated with the sensory recognition of sexual stimuli, as well as the integration and processing of the sensory information of the genitals.

The activation areas of the limbic system are known to be largely associated with the emotional mechanism of sexual stimuli. In this study, the thalamus and amygdala of the limbic system were activated. The activation of the thalamus was commonly observed in previous studies that used visual sexually arousing stimuli [23, 30–33]. These studies reported that the thalamus received visual stimulus information and sent it to a certain area of the cerebral cortex. Further, some studies showed that the thalamus acted as a bridge for transmitting visual information and was also related to the emotional mechanism of sexual arousal [34, 35]. They suggested that the activation of the thalamus is associated with the emotional excitement accompanied by sexual arousal. The amygdala, which was activated together with the thalamus, is also linked to the emotional mechanism of sexual arousal. The usual understanding of the activation of the amygdala is that the amygdala receives various sensory information and transmits the processed information to the dorsal striatum, thalamus, brain stem, prefrontal cortex, and anterior cingulate gyrus. In this process, the amygdala is reported to be associated with the assessment of the emotional aspects (i.e., the degree of pleasure) of complicated sensory information related to sexual stimuli [23, 36]. The amygdala is activated when a sexual stimulus is presented, which results in a large amount of projection to the anterior cingulate gyrus.

Then, the anterior cingulate gyrus is activated, and it is known to be associated with the motivational mechanisms of sexual arousal [7, 10, 23, 36–39]. According to the previous studies, the ACC is known to evaluate the emotional and motivational aspects of input information and to control emotional responses [7, 10, 23, 36–39]. In this respect, the activation of the anterior cingulate gyrus observed in this study suggests the internal conflict between the arousal for the behavioral expression of sexual arousal and the effort to suppress it due to the experimental circumstances. McGuire et al. [40] also pointed out that the orbitofrontal cortex and insular relate to the motivational aspect of sexual arousal, which leads to direct behaviors toward the goal. The orbitofrontal cortex is known to be associated with the prediction of future rewards (i.e., the expectation for the reward as the result of a target behavior) [41].

Other areas activated in this study include the putamen, globus pallidus, and precentral gyrus. These areas are also thought to be associated with the physiological mechanism of sexual arousal. The precentral gyrus is a primary motor area, and it is associated with the control of the self-motion of the penis as well as the imagination of sexual behavior during sexual arousal while a visual stimulus is presented [12, 32, 42]. In particular, the putamen and globus pallidus were not usually activated in the previous block design studies on sexual arousal [8, 22, 38, 39, 43], but their activation was clearly observed in this study. This is believed to be associated with the hemodynamic responses of the putamen or globus pallidus. That is, the neural activity of the putamen and globus pallidus showed more transient aspects of the hemodynamic responses relative to the neural activity of other areas. This may be the one reason why these areas were not activated in the previous block design studies. Globus pallidus and putamen included in the paleostriatum and neostriatum, respectively. The striatum were divided into two subregions such as ventral and dorsal striatum. The function of the dorsal striatum on sexual arousal has been traditionally associated with the penis enlargement [7, 23, 43]. Significant correlation was observed between the level of activity in the putamen and the magnitude of penile erection [7, 23, 43]. Similarly, some animal research also reported that the penis enlargement was shown when an electric stimulus was applied or the bicucullin was injected to stimulate the putamen [44, 45]. Additionally, the activity of the ventral striatum was found to be associated with the subjective level of sexual arousal [13, 23]. Two main functions of this area can be suggested. Firstly, the ventral striatum has been implicated in the reward in that sexual stimuli are perceived as rewards [13, 46, 47]. Secondly, activation of the ventral striatum has been linked to the incentive components of sexual stimuli and to expected reward [38]. In the study, activation of the globus pallidus and putamen in the striatum was reflected in the physiological and reward aspects of the sexual stimuli.

The limitations of this study include the following. First, only heterosexual male subjects are participated in this study, and future studies should examine individuals of various sexual orientations and females. Second, we measured the degree of sexual arousal using the self-report instrument, and the objective measurement of sexual arousal (i.e., the measurement of penis enlargement) should be made in future studies. Third, further research needs to be done to verify whether the difference from the results of previous studies came from the adoption of a different experiment design (i.e., event related or block) or the fMRI protocol (i.e., TR and TE). Fourth, we could not verify that the participant returned to their baseline states within 7–13 s of inter-stimulus interval. In other words, we could not validate this period was enough for young male participants to recover from the sexually aroused state, particularly in terms of the physiological state. The transition between normal and sexually aroused states (emotional and physiological) is relatively slow, and it is quite challenging to go back and forth between two states in the event-related design.

Despite the above limitations, this study successfully induced sexual arousal using visual stimuli. Then, the activated areas were designated as a brain region of interest to check the development of the hemodynamic responses of the region with time. This development is meaningful in that the activation of brain areas which were not frequently observed in the block design studies was identified. In the further study, we are to investigate the physiological response during sexual arousal. The results help us develop and expand the sexual-specific physiological responses in addition to the brain activation involved in sexual arousal. In the further study, if the physiological responses of sexual arousal are included, the findings could contribute to anthropology by revealing human sexual response characteristics of a healthy group.
